# Targeted Nanomedicine to Treat Bone Metastasis

**DOI:** 10.3390/pharmaceutics10040205

**Published:** 2018-10-25

**Authors:** Isaac M. Adjei, Madison N. Temples, Shannon B. Brown, Blanka Sharma

**Affiliations:** J. Crayton Pruitt Family Department of Biomedical Engineering, University of Florida, Gainesville 32611, FL, USA; adjeii@ufl.edu (I.M.A.); mtemples@ufl.edu (M.N.T.); shannonbbrown@ufl.edu (S.B.B.)

**Keywords:** drug delivery, bone marrow, passive targeting, active targeting, nanoparticle

## Abstract

Bone metastases are common complications of solid tumors, particularly those of the prostate, breast, and lungs. Bone metastases can lead to painful and devastating skeletal-related events (SREs), such as pathological fractures and nerve compressions. Despite advances in treatment for cancers in general, options for bone metastases remain inadequate and generally palliative. Anticancer drugs (chemotherapy and radiopharmaceuticals) do not achieve therapeutic concentrations in the bone and are associated with dose-limiting side effects to healthy tissues. Nanomedicines, with their tunable characteristics, have the potential to improve drug targeting to bone metastases while decreasing side effects for their effective treatment. In this review, we present the current state of the art for nanomedicines to treat bone metastases. We also discuss new treatment modalities enhanced by nanomedicine and their effects on SREs and disease progression.

## 1. Introduction

Cancer metastasis is a complex and multistep process in which tumor cells spread from their primary site to distant organs. This process involves the loss of intercellular adhesion, cell migration, angiogenesis, access to systemic circulation, survival in circulation, evasion of the immune response, and growth at a distant organ [1]. In solid malignancies, metastatic spread and systemic disease account for approximately 90% of all cancer-related deaths [2,3], with few improvements in the five-year survival rate over the past decade [2]. Metastases to the bone are common in prostate, breast, and lung cancers, the incidence of which increases with the stage of diagnosis (Table 1) [3,4], and are associated with pain and bone breakages.

The symptoms of bone metastases often are the first sign of disseminated disease in breast and prostate cancer [6,7]. Bone metastases are rarely silent, with 75% of patients experiencing severe pain at the time of diagnosis [6]. Bone metastases can lead to skeletal-related events (SREs), such as pathological fractures, hypercalcemia, and spinal-cord compression, and are associated with shortened survival, decreased quality of life, and increased medical care cost [8]. Current treatments of bone metastases are palliative and aimed at managing SREs and improving patient quality of life. Newer targeted treatments for bone metastases, like nitrogen-containing bisphosphonates and the monoclonal antibody denosumab, have been very efficient at reducing and preventing SREs in patients but are associated with significant side effects, such as renal toxicity and hypocalcemia [8].

Recently, nanotechnology has emerged as a tool to enable early disease diagnosis and for drug delivery to improve disease response and patient quality of life. Nanomedicines approved for clinical use such as Abraxane, an albumin nanoparticle (NP) delivering paclitaxel (PTX), and Doxil, a liposomal formulation of doxorubicin (Dox), improve clinical outcomes and decrease toxicity compared to their corresponding free drugs [9,10,11]. As such, there is significant interest in the application of nanotechnology to improve the outcomes for cancer patients with advanced disease (Table 2). In this review, we present the current state of the art for drug delivery to bone metastases. We also discuss new strategies aimed at improving drug delivery to the bone and their effects on SREs and metastatic disease progression. 

## 2. Treating Bone Metastasis

**Figure 1:**
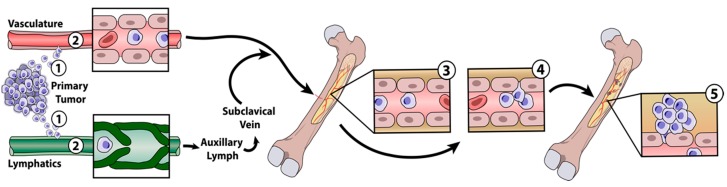
Mechanism of bone metastasis. (**1**) Cancer cells from primary tumor detach and invade surrounding tissue before (**2**) intravasation into the circulatory and lymphatic systems. Lymph, and the cancer cells within it, ultimately enters the bloodstream through the subclavian vein, via the thoracic ducts. Cancer cells that evade the immune system (**3**) translocate through capillaries in the bone where they (**4**) extravasate into the bone marrow to (**5**) establish metastatic sites.

Metastasis to bone is a multistep process, with each step required for the initiation of distant metastatic sites. To metastasize, cancer cells must detach from the primary tumor, (1) invade the surrounding tissue, (2) intravasate into the circulatory and lymphatic systems, evade attack from the immune system, (3) survive and translocate through the bloodstream to bones, (4) extravasate and survive in the bone marrow, and (5) eventually colonize to form a macroscopic secondary tumor in the bone (Figure 1) [19,20,21]. As such, the metastatic process is very inefficient, and few of the tumor cells that start the process develop into bone metastases [22]. Each step in the metastatic process is, conceivably, a drug target to prevent metastasis. For example, ongoing efforts are aimed at preventing the epithelial–mesenchymal transition (EMT) associated with the initial invasion of the surrounding tissues. Other efforts are aimed at modulating the premetastatic niche that recruits tumor cells to secondary tumor sites. To appreciate the role of nanomedicine in metastasis treatment, it is important to have an understanding of current therapeutic options and their drawbacks.

### 2.1. Targeting EMT

The transdifferentiation of cancer cells from an epithelial phenotype to a more motile and invasive mesenchymal phenotype in the EMT process is an important step in metastasis [23] and is also associated with resistance to chemotherapy [24]. Cues from the tumor microenvironment, such as transforming growth factor (TGF) β and epidermal growth factor (EGF), promote EMT initiation and represent potential targets to disrupt the process [25]. As such, inhibitors to TGFβ and EGF cell-surface receptors are under investigation to treat different cancers. Pharmacologic small-molecule inhibitors to TGFβ receptor I, such as LY36497 and SD208, prevent TGFβ induction of EMT in hepatocarcinoma and ovarian cancer [26,27]. The EGF receptor small-molecule inhibitors erlotinib and gefitinib similarly inhibit EMT in breast and lung cancers [28,29]. However, drug resistance due to mutations in these cell-surface receptors makes targeting their intracellular signal transduction pathways necessary to prevent EMT. For example, TGFβ and EGF induce EMT through the Janus kinases/signal transducer and activator of transcription 3 (JAK/STAT3) signaling pathway. Small-molecule inhibitors stattic and AG490 inhibit STAT3 and JAK, respectively, thus inhibiting EMT induction [30,31]. Epithelial-mesenchymal transition is necessary for many processes, such as wound healing, and inhibiting it using systemic therapy could cause substantial side effects [32].

### 2.2. Modulating the Premetastasis Niche

The bone microenvironment is actively modified to support metastatic spread by the primary tumor. Tumors produce soluble factors, including vascular endothelial growth factor (VEGF) and TGFβ, to generate a premetastasis niche (PMN) to support cancer-cell localization and growth, and initiation of metastatic sites [33,34]. Tumors also produce exosomes that contain proteins, mRNA, microRNA, and DNA which home to and regulate cell functions in the PMN [35,36]. Although the PMN in bone metastasis is less studied, it could involve crosstalk between the primary tumor and bone-marrow cells that enhance the CXC chemokine ligand 12 (CXCL12)–CXC chemokine receptor 4 (CXCR4) signaling axis [37]. This environment is a strong chemoattractant to VEGF receptor positive (VEGFR1^+^) myeloid cells and, subsequently, cancer cells to initiate metastasis [38,39,40]. As such, blocking the action of VEGF or exosome-mediated communication between the primary tumor and the PMN could prevent metastasis. The small-molecule inhibitor TSU68 that targets VEGFR2, platelet-derived growth factor receptor β, and fibroblast growth factor receptors 1, modulated the PMN and decreased the incidence of metastasis in animal models of colon cancer [41]. Although promising, most patients are diagnosed with established metastasis, thus making modulating the PMN very difficult.

### 2.3. Treating Established Metastasis

Although there are considerable preclinical efforts to prevent metastasis, patients usually present with metastatic disease at diagnosis. The crosstalk between the tumor cells and bone cells can create an environment that supports osteoblastic or osteolytic metastases [1]. Metastasis diagnosis is based on laboratory screening (calcium, phosphorus, 25-hydroxyvitamin D, alkaline phosphatases, creatinine, thyroid-stimulating hormone, N-telopeptide, and parathyroid hormone levels) [1,7] or imaging (X-ray, bone scintigraphy, computed tomography, magnetic resonance imaging, and positron emission tomography) [42,43,44]. Management of established bone metastasis is complicated and requires a multidisciplinary approach. Depending on the stage of the disease, the extent of skeletal involvement, prior treatment history, and the overall health of the patient [1], disease management includes a combination of surgery, radiotherapy, chemotherapy, hormone therapy, bisphosphonates, and immunotherapy [45].

#### 2.3.1. Surgery

Surgical treatment is an option for localized bone metastasis, but is not applicable for advanced disease with multiple metastatic sites. Surgery for bone metastasis is palliative and aims to alleviate pain [46] or to secure weight-bearing bones at a high risk of fracture [47]. Supplementing the surgical treatment of metastases with other treatment modalities, such as radiotherapy and chemotherapy, can improve the clinical outcomes [7]. However, surgical-site infections, recovery time, and other surgical complications may outweigh the potential clinical benefits of decreased pain and improved function [47]. Moreover, not all patients benefit from reduced pain and increased functionality after surgery [47]. Surgical management of metastases has been an area of controversy due to the lack of conclusive data regarding improvements to the quality of life, pain, and functional outcomes, as well as the associated complications and mortality rates [47,48]. 

#### 2.3.2. Chemotherapy

Systemic anticancer chemotherapy helps manage primary tumors and bone metastases. Taxanes (docetaxel and cabazitaxel), which are mitotic inhibitors, remain the only chemotherapeutic options for patients with advanced-stage prostate cancer [49]. Treatments for breast cancer rely on anthracyclines (inhibitors of DNA and RNA synthesis, free-radical generators), taxanes, vinorelbine (microtubule disruptor), capecitabine (thymidylate synthase inhibitor), and platinum agents (DNA crosslinker), particularly in patients with hormone-insensitive tumors or after patients become refractory to hormonal therapy [50]. Chemotherapy is the primary treatment option for patients with systemic disease, but is associated with toxicity and side effects, including febrile neutropenia [51], hypersensitivity reactions [52], and cardiotoxicity [53]. Due to these toxic effects, many men with metastatic prostate cancer are deemed too ill to be eligible for docetaxel treatment [54]. In fact, in such cases, docetaxel treatment actually decreases survival [55].

#### 2.3.3. Radiotherapy

Radiotherapy for bone metastases uses external-beam radiotherapy or radiopharmaceuticals to induce DNA damage and cause apoptosis of tumor cells [56]. External-beam radiotherapy uses a proton beam or high energy gamma radiation to kill the cancer cells and has been shown to relieve bone pain within 14 days of treatment and decrease fractures in patients [57]. External-beam radiotherapy is limited for treating multifocal bone metastases. Although whole-body beam therapy is used in some patients, particularly those with multifocal metastases, a low dose of radiation is used to decrease the risk of bone-marrow suppression, which can cause infections and anemia [58].

Radiopharmaceuticals are used to treat patients with extensive bone metastases where external beam therapy is not practical. Phosphorous-32 (^32^P), strontium-89 (^89^Sr), and rhenium-186 (^186^Re) etidronate are bone-seeking radiopharmaceuticals that accumulate preferentially in osteoblastic bone metastases compared to normal bone due to an increased rate of bone formation [59,60]. Radiopharmaceuticals are useful tools for palliating pain; however, when used alone, they do not contribute to a significant overall survival benefit [61]. Myelotoxicity, which causes low blood-cell counts, is a side effect of radiopharmaceutical use. Since these radiopharmaceuticals show affinity to bone formation, their application in metastases with an osteolytic phenotype is limited.

#### 2.3.4. Hormone Therapy

Prostate- and breast-cancer subtypes depend on hormones for growth and development, and hormone therapy is a cornerstone for patients with hormone-receptor positive tumors. The use of hormone-receptor antagonists (e.g., tamoxifen), orchiectomy, and castration alleviates pain in 70% of treated patients and these remain vital tools for managing bone-only or bone-predominate metastases [62]. Ultimately, tumors become resistant to hormone therapy by producing hormones themselves or becoming hormone insensitive, and are termed castrate-resistant. In castrate-resistant prostate tumors that produce hormones, treatment with abiraterone, an inhibitor of steroid 17-α-hydroxylase/17,20 lyase, a critical enzyme in testosterone biosynthesis [62], is preferred. This treatment has been shown to increase radiographic free progression by eight months when used in combination with prednisone compared to prednisone alone [63]. However, hormone therapy has associated side effects, with some patients experiencing loss of sex drive, impotence, and osteoporosis [64].

#### 2.3.5. Bisphosphonates

Bisphosphonates are analogs of pyrophosphates present in the bone matrix and they have a high affinity and selectivity to hydroxyapatite (HAp) [65,66,67]. Bisphosphonates are common treatments for skeletal diseases such as Paget’s disease of the bone and osteoporosis. In bone metastasis patients, bisphosphonates decrease the frequency of SREs by 30–40% [68]. There are two classes of bisphosphonates, the simple non-nitrogen-containing (e.g., clodronate, etidronate, tiludronate), and the nitrogen-containing (e.g., alendronate (Aln), zoledronate (Zol), pamidronate, risedronate, ibandronate) [69]. Bisphosphonates demonstrate varying affinities to bones, in the order pamidronate > alendronate > zoledronate > risedronate > ibandronate [70]. Bisphosphonates may also slow tumor growth [71] by preventing growth-factor release from the bone as a result of osteoclast-function inhibition [72]. Although effective at preventing SREs, chronic bisphosphonate use is associated with osteonecrosis of the jaw [73] caused by inhibiting the role of endothelial progenitor cells in angiogenesis [74]. Additionally, chronic intravenous use of bisphosphonates can also result in renal failure [73].

#### 2.3.6. Denosumab

Denosumab is a fully human, monoclonal, synthetic antibody that inhibits receptor activator of nuclear factor-κβ ligand (RANKL) with high affinity, thereby blocking the development of osteoclasts [71]. Soluble RANKL is released into the bone microenvironment where it binds to and activates its receptor RANK on immature osteoclasts, acting as a critical factor for osteoclast differentiation and activation. The monoclonal antibody denosumab binds RANKL and prevents bone resorption by inhibiting both mature osteoclast function and osteoclast differentiation, thus interrupting the vicious cycle of bone destruction [75].

Denosumab is indicated for the prevention of SREs and is just as effective as Zol in delaying the time to first SRE by 8.2 months, reducing the risk of a first SRE by 17%, and reducing the risk of multiple SREs by 18% [76]. Denosumab reduced the risk of fractures without an increase in the risk of delayed fracture healing, hypoglycemia, or osteonecrosis [77]. However, there is no difference in overall patient survival between treatment with denosumab or zoledronic acid [75,76].

## 3. Targeting Nanoparticles to Bone Metastasis

Advances in the treatment of bone metastasis have reduced the pain and incidence of fractures associated with the disease [78]. Unfortunately, challenges remain as many potential drugs for treating metastases do not reach the bone in efficacious concentrations. Increasing the dosage of drugs in order to achieve therapeutic concentrations in the bone results in toxicity that may not be tolerated by severely ill patients [54]. Drugs that reach well-vascularized primary tumors may not accumulate in metastases that are poorly vascularized. To compound issues, the dense extracellular matrix associated with osteoblastic bone metastases decreases drug penetration and accumulation into the metastatic site, and reduces drug uptake into cancer or other target cells [78,79]. This complex problem calls for new strategies to target therapies to bone metastases for effective treatment and minimal systemic toxicity.

Nanoparticles (NPs) serve as carriers for small-molecule drugs, proteins, and nucleic acids to treat bone metastasis [80]. Due to their versatility, NPs have been used to deliver molecules for remodeling blood vessels, increasing oxygen tension, or augmenting immunity to improve cancer treatment [81,82,83,84]. Therapeutics loaded into or conjugated to NPs are protected from degradation, have improved pharmacokinetics, and can be localized to specific disease locations. Loaded or conjugated therapeutics can be released depending on site-specific physiological characteristics or via external stimuli, such as an alternating electromagnetic field, ultrasound, or light [82]. Nanoparticles thus increase drug efficacy, decrease off-target effects, and lower the amount of administered drug that directly decreases toxicity.

Nanoparticles also improve the efficacy of other modalities currently in practice to treat bone metastasis. Particles made from metals and metal oxides can generate heat when stimulated by external stimuli, such as ultrasound, radiofrequency, and magnetic fields, leading to the thermal ablation of tumors [85]. Nanoparticles also enhance photothermal therapy by improving the localization of photosensitizers to tumors [85,86,87]. The application of nanotechnology to improve current treatment modalities is discussed in detail in a later section of this review.

The effectiveness of NPs is dependent on their accumulation in vascularized solid tumors via the enhanced permeability and retention (EPR) effect. Fast-growing tumors develop poorly organized vasculature with large endothelial fenestrations that allows NPs to accumulate in the tumor interstitium. Tumors also have impaired lymphatic drainage that enhances NP retention in the tumor [88,89]. Although the EPR effect has served as the foundation for most cancer nanomedicines, there is heterogeneity in its presentation based on tumor type and patient population. Furthermore, the size of a tumor and the heterogeneous tumor microenvironment affect the EPR effect [90]. The existence of the EPR effect in metastasis is an unresolved question, with studies reporting that metastases exhibit the phenomenon [91], while others report otherwise, especially in early-stage metastasis [92].

Targeting of therapeutics to bone metastases or any other tissue can utilize the unique physical characteristics of the tissue (passive targeting) to improve drug delivery. In the bone, passive targeting takes advantage of the fenestrations in bone-marrow capillaries. Active targeting exploits specific receptors upregulated on bone-marrow endothelial cells or specific interactions with the bone matrix to improve drug delivery. In this section, we discuss the different methods of targeting, the ligands used, and the NPs utilized (Table 3).

### 3.1. Passive Targeting

**Figure 2:**
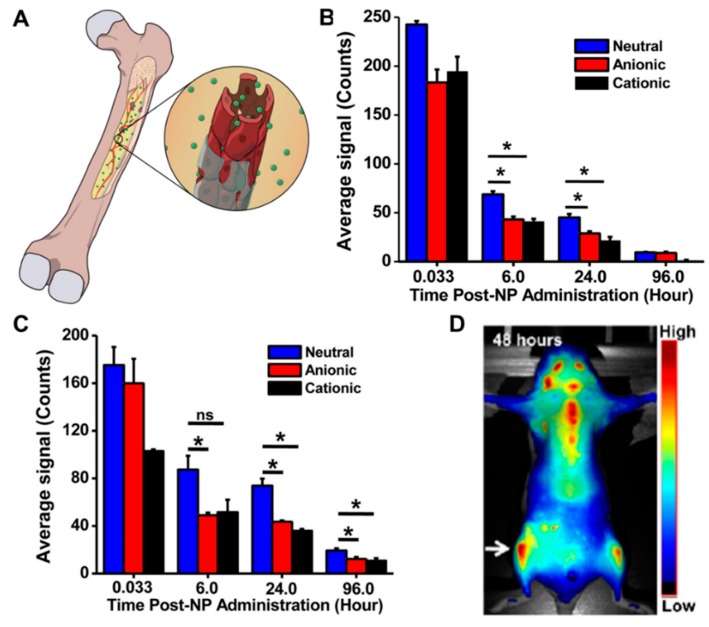
Passive targeting of nanoparticles to bone marrow. (**A**) Scheme showing passive targeting of NPs (green spheres) into bone. NPs pass through the fenestrations in sinusoidal capillaries to localize in bone marrow. NP (**B**) charge on blood residence time and (**C**) bone accumulation. Neutral charge NPs showed greater accumulation in tibia than similarly sized anionic and cationic NPs. (**D**) Neutral charge NPs showed greater localization in tibia with bone metastasis (white arrow) than healthy tibia. Reprinted with permission from Reference [93].

The bone marrow contains highly fenestrated capillaries with pore sizes up to 170 nm in diameter [93]. These clefts can be exploited to increase drug delivery into the bone marrow (Figure 2A). We have previously demonstrated that NPs smaller than the size of the bone-marrow fenestrations accumulate in the bone-marrow space [93]. Interestingly, the extent of NP accumulation in the bone was dependent on their ability to evade the reticuloendothelial system and remain in circulation, thereby allowing the NPs sufficient opportunity to pass through the sinusoidal capillaries. In these studies, neutrally charged poly (lactic-co-glycolic acid) (PLGA) NPs had higher blood half-life and showed greater accumulation in bone than similarly sized anionic and cationic PLGA NPs, underscoring the impact of NP charge on biodistribution (Figure 2B,C). Interestingly, the NPs localized in early-stage metastases within the bone marrow that were not associated with the bone (Figure 2D). Therefore, nanoparticles designed to target only the mineralized bone matrix may not be effective in targeting these early metastases within the bone marrow [93]. 

The use of monodisperse polystyrene NPs has provided further insight into the effect of the physical characteristics of NPs on their localization in bone marrow. Less than 1% of the injected dose of bare polystyrene NPs (diameters of 60 nm and 150 nm) localized to the bone marrow [100]. Coating the 60 nm and 150 nm polystyrene NPs with the hydrophilic polymer poloxamer 407 increased their blood residence time and improved their bone-marrow accumulation four-fold. However, for 250 nm polystyrene NPs, bone-marrow accumulation remained below 1% even after coating with the hydrophilic polymer, demonstrating the impact of size on accumulation [100]. The importance of size is further underscored by the accumulation of 16 nm trifolium-like platinum nanoparticles [94] and 50 nm silica NPs [103] in the bone marrow. These studies demonstrate that NPs must have high blood residence time and a diameter less than 150 nm to accumulate in the bone marrow (Figure 2). These results confirm the relationship between the physical characteristics of NPs and their pharmacokinetics and bone-marrow localization. 

### 3.2. Active Targeting

Active targeting of therapeutics to bone metastases takes two main forms: direct targeting to the bone matrix using bisphosphonates or targeting therapeutics to the bone marrow by using ligands that interact with the receptors of endothelial cells lining the capillaries. Once nanoparticles accumulate within the bone, they may be further targeted to specific cells for drug delivery. Depending on the type of treatment, cellular targets can include the cancer or stromal cells (fibroblasts, mesenchymal stem cells, macrophages, or T-cells) [104].

#### 3.2.1. Direct Targeting to Bone

Although bone metastasis initially starts in the bone marrow and associates with the mineralized bone in later stages, most NP design strategies for treating bone metastasis have targeted the mineralized bone. Mineralized bone is composed of HAp, which accounts for 50% of bone volume and 70% of bone weight [67]; HAp is thus a logical target for bone drug delivery.

##### Bisphosphonates

The high selectivity and affinity of bisphosphonates to bone make them useful for drug delivery to bone metastases. In addition to binding to the bone, bisphosphonates have demonstrated induction of apoptosis in cancer cells by indirect inhibition of adenine nucleotide translocase via intracellular accumulation of isopentenyl pyrophosphate [105]. Bisphosphonates also improve tumor response to treatment by depleting tumor-associated macrophages that support tumor growth. These additional properties make bisphosphonates attractive for targeted drug delivery to sites of bone metastasis [67].

**Figure 3:**
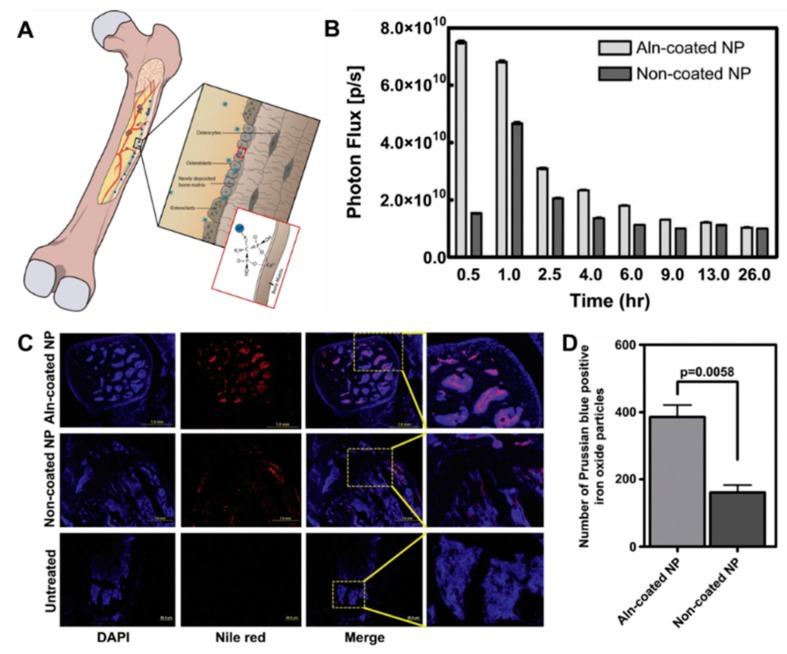
Active targeting of nanoparticles to bone using bisphosphonates. (**A**) Scheme showing targeting of bisphosphonate-functionalized NPs to bone. (**B**) Quantification of alendronate (Aln)-conjugated NPs accumulation into bone over time. (**C**) Representative image of Nile red-labelled NP localization in mouse femurs. (**D**) Quantification of number of NPs in the bone marrow. Reprinted with permission from Reference [96].

Various bisphosphonates, particularly Aln and Zol, have been explored for active targeting of nanoparticles to the bone. The presence of a primary amine in Aln and the modifiable nitrogen group in Zol makes them amenable to common conjugation chemistries used with drug-delivery systems [65,67]. As such, bisphosphonates have been used to target different types of NPs, including polymeric, inorganic, and liposomes, to the bone matrix (Figure 3). 

Conjugating, anchoring, or coating of NPs with bisphosphonates increased the accumulation and retention of polymeric biodegradable NPs in bone tumors [96]. Conjugation of Aln to NPs, formulated from block copolymer PLGA and polyethylene glycol (PEG) (PLGA-b-PEG), showed a six-fold increase of NP accumulation into bone [95]. Improvements in bone accumulation were observed with other Aln-conjugated polymeric nanocarriers, which corresponded to successful delivery of therapeutics to the bone after intravenous injection [95,96,97] (Figure 3B–D). The conjugation of Zol to PLGA-PEG NPs increased their accumulation into the bone by 50-fold after 24 hours postinjection. Importantly, the amount of Zol-conjugated PLGA-PEG NPs in bone metastases was five-fold greater than in naïve bones, suggesting increased specificity to the lesions [97]. An important aspect of bisphosphonate-mediated targeting is the decrease in toxicity attributed to changes in drug pharmacokinetics and biodistribution. For example, Aln-conjugated micelles decreased cardiotoxicity of doxorubicin and increased efficacy at inhibiting bone resorption in animal models of bone metastasis [99]. 

Bisphosphonates also readily bind onto inorganic NPs for drug delivery and imaging applications. For example, Zol-anchored mesoporous silica NPs demonstrated four-fold greater binding to bone compared to plain silica NPs. Additionally, Zol-coated mesoporous silica NPs showed more significant interactions with cancer cells, resulting in higher cell death [106]. 

The addition of imaging functionality to the NPs can improve their clinical application by improving diagnostic techniques or enabling tracking of therapeutic particles in the body. Zoledronate-anchored mesoporous silica NPs covered with gadolinium have been employed for imaging bone metastases, in addition to treating them [98]. In these studies, Zol is anchored to the silica NPs by the pH-sensitive linker *N*,*N*-carbonyldiimidazole, to ensure the release of Zol after cellular endocytosis of the NPs, to improve its action intracellularly [98].

Similarly, self-assembling pH-sensitive micelles functionalized with PEG showed increased accumulation into bone metastasis after conjugation to Aln. Once in the acidic tumor microenvironment, the NPs degrade to release their therapeutic load [99]. 

##### Tetracyclines

Tetracyclines (TETs) are natural or semisynthetic broad-spectrum antibiotics (acting on both gram-positive and gram-negative bacteria) that inhibit protein synthesis in bacteria [107]. The affinity of TETs to bone was observed soon after their discovery [108]. Tetracyclines have high affinity to HAp and inhibit osteoclast function, which resulted in their disuse in pediatric medicine, as they may prevent bone growth and remodeling [109].

The high affinity of TET to HAp can be exploited to target NPs to the bone, while the innate fluorescence of TET under ultraviolet light allows for the rapid assessment of the targeting efficiency of TET-conjugated NPs [110]. The conjugation of TET to PLGA NPs increased bone accumulation two-fold compared to untargeted NPs. Interestingly, TET-conjugated NPs also had lower accumulation in the liver, suggesting a general change in the biodistribution and pharmacokinetics of the NPs [101].

##### Peptides

Hydroxyapatite-binding peptides provide another strategy for localizing therapeutics to the bone. Early studies mimicked the repeating acidic amino acid sequences of non-collagenous HAp-binding proteins by using repeating units of aspartate [111] or glutamic acid [112] to increase bone localization. Subsequent studies, however, utilize phage display to identify unique peptides that bind to HAp [113,114]. Ultimately, these peptides could be utilized with various polymeric, inorganic, or liposomal NPs to target the bone lesion. 

#### 3.2.2. Active Targeting to Bone-Marrow Endothelium

**Figure 4:**
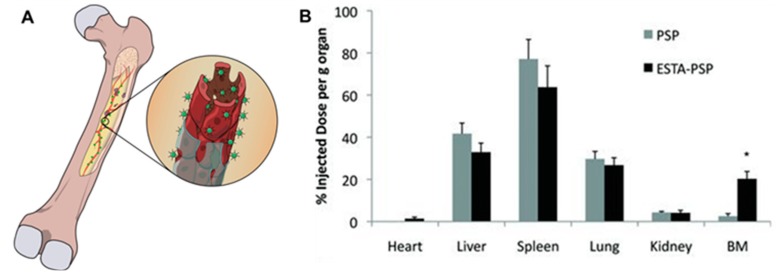
Drug delivery to the bone-marrow vasculature. (**A**) Scheme showing NP interaction with endothelial cells of bone-marrow capillaries. (**B**) Accumulation of E-selectin-targeted NPs (ESTA-PSP) to bone. Reprinted with permission from Reference [102].

Drug delivery to the bone marrow is a promising approach to treat early-stage bone metastasis when the secondary tumor in the bone has not ossified and remains in the marrow space. Strategies for bone-marrow localization of NPs take advantage of the unique characteristics of the endothelial cells of bone-marrow capillaries. Endothelial cells that line the bone-marrow capillaries constitutively express E-selectin and vascular cell adhesion molecule-1 that enables cell homing [115]. Conjugation of E-selectin-binding aptamers to NPs has been explored to increase NP localization into the bone marrow (Figure 4). Recently, a thioaptamer with nanomolar affinity for E-selectin was identified to improve NP accumulation into the marrow space [116]. Conjugation of this E-selectin-binding thioaptamer to porous silicon NPs increased NP accumulation into the bone marrow eight-fold compared to nontargeted NPs [102]. The clinical relevance of this targeting approach was confirmed by the accumulation of paclitaxel loaded liposomes into the bone marrow with high efficiency after conjugation with the E-selecting-binding thioaptamer [102]. 

#### 3.2.3. Active Targeting to Tumor Cells in Bone Marrow

Cancer cells within the bone are target options because of their upregulation of certain cell-surface markers, including cadherin-11 and αvβ3 integrin. Taking advantage of the upregulated expression of αvβ3 integrin in breast-cancer cells in the bone microenvironment, one group developed micelle-based NPs functionalized with a targeting ligand for αvβ3 integrin and loaded the particle with docetaxel. These NPs localized to the bone metastases in a metastatic breast-cancer murine model, reducing the tumor burden with less bone destruction and toxicity compared to the free docetaxel [117]. 

## 4. Using Nanomedicine to Improve Current Therapy Regime for Bone Metastasis

Nanomedicine has improved the efficacy of chemotherapeutic drugs for bone metastasis while decreasing toxicity. The application of nanotechnology has also introduced new therapeutic regimens based on nucleic acid delivery and photothermal therapy that hitherto may not have been possible.

### 4.1. Chemotherapy

Nanoparticle-mediated delivery of chemotherapy improves tumor targeting while minimizing toxicity. Doxorubicin (Dox) is a potent first-line therapy for bone metastasis, but it induces cardiotoxicity. Loading Dox into PLGA NPs decreases cardiotoxicity and improves drug pharmacokinetics, resulting in better response to therapy [118,119]. Doxorubicin loaded into Aln-conjugated PLGA NPs decreased the incidence of bone metastasis and the number of osteoclasts in the metastasis, while decreasing overall toxicity [120]. The tolerance of experimental animals to Dox was further improved by functionalizing pH-sensitive groups to NPs to induce Dox release in the acidic tumor microenvironment, thereby improving the specificity of targeting while decreasing toxicity [99]. Similarly, PLGA NPs successfully deliver paclitaxel (PTX) to bone metastases and preserve the bone [93,121]. In an intraosseous prostate-cancer model of bone metastasis, neutrally charged PLGA NPs loaded with PTX were successful in decreasing tumor growth and preventing bone loss [93]. These PTX-loaded NPs were well-tolerated, as injected mice did not lose weight compared to paclitaxel in cremophor [93]. In studies using Aln-conjugated *N*-(2-hydroxypropyl) methacrylamide (HPMA) copolymer to deliver PTX to treat bone metastases, tumor growth and microvessel density were significantly reduced compared to untargeted controls, without systemic toxicity. Critically, bone preservation was achieved by Aln-conjugated HPMA-mediated delivery of PTX [122,123]. 

The potential for NPs to provide combination drug therapy, such that multiple drugs that target different pathways in cancer progression are loaded into a single NP system, is appealing for the treatment of bone metastasis. For example, codelivery of curcumin (an inhibitor of osteoclasts) and bortezomib (a protease inhibitor) by PLGA NPs inhibited the progression of bone metastases and protected against bone loss due to osteoclast activity [96]. In this study, dual curcumin and bortezomib-loaded NPs were more effective at inhibiting tumor growth and osteolysis than NPs loaded with either drug alone [96]. Another excellent application of combination therapy is the codelivery of Silibinin, an inhibitor of EMT, and α-tocopherol, an antioxidant, in solid lipid NPs to decrease the incidence of bone metastasis [124]. 

Inorganic NPs have also been employed to deliver chemotherapeutics to bone metastases with the goal of decreasing osteoclast function. Inorganic mesoporous silica NPs targeted to the bone with Zol efficiently deliver plumbagin, a natural compound that targets the JNK/ERK pathway, to decrease cancer-mediated osteoclast formation, and hence reduce bone loss [125]. To improve site-specific drug release and decrease toxicity, the mesoporous silica NPs were further modified to be pH-sensitive to release plumbagin only in the acidic tumor microenvironment [98]. 

### 4.2. Nucleic Acid Delivery

Dysregulation and lack of epigenetic control of essential genes is a hallmark of cancer progression and metastasis. As such, RNA interference is a powerful tool for suppressing the expression of oncogenes with high specificity to improve patient survival. Similarly, reactivation of tumor-suppressor genes in cancer cells promotes their response to other therapies. A key challenge to the application of nucleic acids as therapy is their rapid degradation by nucleases or their stimulation of the immune system [126,127]. Due to their inherent negative charge and large molecular weights, nucleic acids also show little uptake into target cells, which is critical for their function. Encapsulation or complexation of nucleic acids with different nanotechnology platforms improves stability, decreases immunogenicity, and enhances their delivery to cells of interest. Importantly, these NP systems, which generally are cationic or possess targeting ligands to enhance the uptake of nucleic acids into target cells, are key enabling technologies for the therapeutic use of nucleic acids in cancer [126,127,128]. 

Nanoparticle-mediated delivery of short interfering RNA (siRNA) can inhibit different aspects of the bone metastasis process after both local and systemic injection. Local injection of PLGA NPs loaded with siRNA against osteopontin and bone sialoprotein decreased the incidence of bone metastasis and bone lesion size compared to free siRNA delivered via an osmotic pump in an intraosseous bone-metastasis model. Interestingly, even at a lower dose, siRNA in NPs showed greater uptake and results in greater knockdown of the osteopontin and bone sialoprotein genes compared to free siRNA [129]. In addition to improving gene knockdown, NP-mediated delivery of siRNA protects the siRNA from immune attacks. Atelocollagen-mediated delivery of siRNA decreased siRNA immunogenicity and reduced the systemic production of interleukin 12 or interferon gamma after intravenous injection compared to free siRNA [130]. Complexing of siRNAs targeted to the zeste homolog 2 and phosphoinositide 3′-hydroxykinase p110-α-subunit to atelocollagen effectively knockdown the genes and decrease the incidence of bone metastases after intracardiac injection of bone metastatic prostate-cancer cells [130]. 

In addition to siRNAs, NPs can be used for the delivery of tumor-suppressor microRNAs (miR) to downregulate oncogenes. Chitosan NP-mediated delivery of miR-34a downregulates the expression of the proto-oncogenes MYC, MET, and AXL, and induces autophagy in cancer cells [131]. Nanoparticle-mediated delivery of miR-34a to bone metastases also downregulates transforming growth factor-β-induced factor 2 in cancer cells, thereby inhibiting osteoclast formation and subsequent bone resorption [132]. Similarly, atelocollagen-mediated codelivery of miR-15a and miR-16-1 prevented invasion of cancer cells into surrounding tissue [133]. Taken together, these studies demonstrate the effectiveness of nanotechnology at introducing nucleic acids to tumors and bone niches to treat and/or prevent bone metastasis. 

### 4.3. Hyperthermia Therapy

Nanoparticles have versatile physicochemical and tumor-selective properties that make them useful for thermal tumor-ablation therapy. In this treatment modality, sufficiently high temperatures are generated within tumors to induce apoptosis by denaturing proteins, fragmenting lipid membranes, and increasing oxidative stress. Nanotechnology-mediated thermal ablation of bone metastasis can be classified as photothermal or magnetic field induced heating—each with its own advantages [85,87,134] 

Gold NPs, carbon nanotubes, and graphene with high photothermal conversion efficiencies, when illuminated with near-infrared light, decreased tumor growth and increased overall survival of tumor-bearing mice [135,136]. Additionally, PEGylated carbon nanotubes decreased the growth of an intraosseous model of bone metastasis after intravenous injection and irradiation with near-infrared light. Tumors in mice injected with the PEGylated carbon nanotubes at the tumor site achieved temperatures greater than 70 °C, while the surrounding tissue maintained ambient temperature [86]. Similar results were achieved with trifolium-like platinum NPs that inhibited tumor growth and prevented osteolysis [94]. However, the limited depth of penetration of the irradiating light, which is exacerbated by bone density, inhibits the widespread application of nanotechnology-based photothermal therapy. As such, other approaches for heating tumors are being explored, including using magnetic NPs in conjunction with alternating magnetic fields [137].

Hyperthermia cancer therapy with magnetic NPs in a magnetic field enables treatment of deep-lying tumors. These therapies may be crucial for prostate cancer where the incidence of metastasis in the vertebrae and pelvic bones is high and not easily treated with photothermal therapy. Hyperthermia therapy in bone is feasible with ferromagnetic bone cement [137,138] or magnetic NPs [139,140]. 

### 4.4. Radiotherapy

Nanomedicines can also decrease the toxicity associated with radiopharmaceutical-mediated radiotherapy. Nanocarriers increase radioisotope localization into tumors and decrease nonspecific accumulation in healthy tissue. Copper sulfide (^64^CuS) NPs accumulate in tumors and irradiate cells to decrease growth and metastasis [141]. The efficacy of ^64^CuS NPs is increased by the incorporation of ^131^Iodine into the particle, which magnifies the amount of ionizing radiation the tumors experience [142]. An advantage of using NPs for radiotherapy is the ability to have multimodality treatments to improve tumor response. For example, ^64^CuS NPs take advantage of ionization radiation from ^64^Cu and the plasmonic properties of the CuS NPs to enable both radiotherapy and photothermal therapy [141]. 

Nanomedicines can also increase the effectiveness of external-beam radiotherapy by acting as radiosensitizers [143]. Gold NPs increase the killing of radiation-resistant cancer cells and improve the survival of tumor-bearing mice [144]. The ability of gold NPs to increase the radio sensitivity of cells is attributed to the arrest of cancer cells in the G2/M phase of the cell cycle [145].

### 4.5. Disease Diagnosis and Detection

Early detection of metastases could improve the treatment options and survival of cancer patients. Circulating tumor cells (CTC) are a precursor to metastasis at distant sites, so their detection is important. Studies of nanobased approaches to identify CTC in peripheral blood that use antibody-conjugated surface-enhanced Raman spectroscopy (SERS) have demonstrated encouraging results [146,147,148]. Enrichment of cancer cells from peripheral blood using magnetic NPs is another strategy [149,150], which can be coupled to photoacoustic tomography for rapid diagnosis [151,152,153]. 

### 4.6. Nanoparticles Targeted to Different Stages of the Metastasis Process

The unique characteristics of nanomedicines give the opportunity to target different aspects of the metastasis process, not just the established metastasis.

#### 4.6.1. Epithelial Mesenchymal Transition

The inherent bioactive properties of nanomaterials can be therapeutic on their own. Unmodified gold NPs reversed EMT in ovarian cancer cell lines by downregulating the expression of mesenchymal markers N-cadherin and vimentin as well as Snail, a transcription factor associated with EMT. The gold NPs reduced the expression of several cytokines, including TGFβ, a potent initiator of EMT [30,154]. Importantly, the gold NPs decrease peritoneal metastasis and the number of metastatic nodule formation [154]. It is possible that gold NPs prevent EMT through mechanisms other than reduced TGFβ expression, but this requires further study. Tumor-specific gelatinase-cleavable PEG and poly(ε-caprolactone) NPs codelivered miR-200c, a downregulator EMT and metastasis [155], and docetaxel in a gastric cancer xenograft model, which enhanced docetaxel cytotoxicity and reversed EMT [156]. This nanoparticle accumulated and was retained in the tumor, and decreased the expression of class III beta tubulin, a target of miR-200c, and ultimately inhibited EMT [156]. 

#### 4.6.2. Targeting Circulating Tumor Cells

Removing CTCs from blood and lymph has the potential to decrease the incidence of metastasis. In a proof-of-concept study, magnetic NPs (MNPs) functionalized with ephrin-A1 mimetic peptides removed ovarian cancer cells overexpressing the EphA2 receptor from peritoneal fluid in a murine model of ovarian cancer. This treatment reduced CTCs with corresponding decrease in metastasis [157]. These techniques of targeting CTCs has been advanced with synthetic silica NPs functionalized with activated platelet membrane, which resulted in their interaction with CTC-associated thrombi in lungs. When TNF-Related Apoptosis Inducing Ligand (TRAIL) was conjugated to these NPs, the incidence of lung metastasis was dramatically decreased in a model of mouse metastatic breast cancer [158].

## 5. Future Perspective and Conclusions

Nanomedicine has advanced therapeutic modalities for bone metastases. Nanoparticles enable tumor-ablation therapy and improve the efficacy of photothermal therapy even in deep bone lesions [135,142]. Various NPs, when used as delivery agents for radioisotopes and chemotherapeutic drugs, increase drug efficacy and decrease toxic side effects by modifying drug pharmacokinetics and biodistribution. Most of the current generation of NPs that target the skeletal system bind to the mineralized bone and have been efficacious in preclinical animal models. However, treatment in these models is initiated before any appreciable bone loss, thus making these therapies effective even in osteolytic bone-metastasis models. Further studies of these drug-delivery systems in animal models of advanced osteolytic disease would elucidate their effectiveness and guide future developments. 

Even with advances in drug-delivery systems, treatments for advanced multifocal metastases remain a challenge and are mostly palliative. As such, early disease diagnosis is needed to ensure prompt treatment. Advances in the nanotechnology-mediated detection of primary tumors or metastatic lesions via different imaging modalities have enabled early disease detection. The use of NPs in liquid biopsies to detect CTCs also allows for prompt disease diagnosis. However, a gap remains in our ability to diagnose micrometastases that do not exhibit the EPR effect used by NPs for tumor accumulation. Moreover, micrometastases may be beyond the detection limits of clinical-grade MRIs. NPs coupled to high-resolution imaging modalities, such as photoacoustic tomography, could enable the detection of micrometastases resulting in early treatment. 

Overall, preclinical studies for treating bone metastases with nanomedicine are encouraging, and show that nanomedicines could provide better treatment options for cancer patients to improve survival and quality of life. 

## Figures and Tables

**Table 1:** Five-year incidence and survival of bone metastases by tumor type [4,5].

Tumor Type	% Incidence of Bone Metastases (95% CI)	% 5-Year Survival (95% CI)
Prostate	24.5 (23.9–25.1)	6 (5 to 7)
Lung	12.4 (12.1–12.7)	1 (0.5 to 1)
Renal	8.4 (8.0–8.9)	5 (4 to 7)
Breast	6.0 (5.8–6.1)	13 (11 to 14)
Gastrointestinal	3.2 (3.0–3.4)	3 (2 to 5)

**Table 2:** Nanomedicines in clinical trials for treating advanced-stage cancers. The targeting strategy for all cases in passive.

Name (Active Drug)	Carrier	Cancer Type	Clinical Status	Reference
Caelyx (doxorubicin)	liposome	Metastatic breast cancer/advanced ovarian cancer	Approved	[10]
NK-105 (paclitaxel)	Polymeric micelle	Metastatic breast cancer	Phase III	[12]
EndoTAG-1 (paclitaxel)	Liposome	Metastatic triple-negative breast cancer	Phase III	[13]
ABI-009 (rapamycin)	Albumin NP	Advanced sarcoma	Phase II	[14]
CRLX-101 (camptothecin)	Polymeric NP	Advanced renal carcinoma	Phase II	[15]
CPX-1 (Irinotecan HCl:Floxuridine)	Liposome	Advanced colorectal cancer	Phase II	[16]
SGT53 (p53 cDNA)	Liposome	Metastatic pancreatic cancer	Phase II	[17]
DepoVax (tumor antigen)	Liposome	Advance-staged breast, prostate, and ovarian cancers	Phase I	[18]

**Table 3:** Targeting strategies and applications of bone-targeted drug-delivery systems.

Carrier	Targeting Strategy	Application	Target	Cancer Type	Outcome	Reference
Poly (lactic-co-glycolic acid) (PLGA) nanoparticles (NPs)	Passive targeting via neutral charge and size (150 nm)	Paclitaxel delivery in intraosseous model of bone metastasis	Cancer cells	Prostate cancer	Slowed metastasis growth and reduced bone loss	[93]
Platinum NPs	Passive targeting via size (15 nm)	Photothermal therapy in intraosseous model of bone metastasis	Cancer cells	Prostate cancer	Prevents tumor growth and inhibits osteolysis	[94]
PLGA-b- polyethylene glycol (PEG) NP	Active targeting using Aln binding to bone	Bortezomib delivery in bone	Cancer cells	Myeloma	Slowed tumor growth and improved survival	[95]
PLGA NPs	Active targeting using Aln binding to bone	Curcumin and bortezomib delivery in intraosseous model of bone metastasis	Cancer cells	Breast cancer	Decreased tumor growth rate and bone resorption	[96]
PLGA–PEG NPs	Active targeting via Zol binding to bone	Doxetaxel delivery in intraosseous model of bone metastasis	Cancer cells	Breast cancer	Increased doxetaxel delivery to bone	[97]
Mesoporous silica-covered gadolinium NPs	Active targeting via Zol binding to bone	Theranostic NPs for bone metastasis imaging and plumbagin delivery in intracardiac cancer cell injection model of bone metastasis	Cancer cells	Breast cancer	Inhibited tumor initiation and osteoclast formation	[98]
Self-assembled PEG micelles	Active targeting via Aln	Doxorubicin delivery in intraosseous model of bone metastasis	Cancer cells	Lung cancer	Decreased cardiac toxicity and reduced bone loss	[99]
Polystyrene NPs	Passive targeting via size (60 nm, 150 nm) and hydrophilicity	Evaluate NP characteristics necessary for bone localization	Not applicable	Healthy animals	Not applicable	[100]
PLGA NPs	Active targeting via tetracycline binding to bone	Delivery of simvastatin	Osteoblasts	Healthy animals	Improved bone density	[101]
Porous silicon NPs	Active targeting to bone marrow capillaries via E-selectin targeting aptamer	Paclitaxel delivery to bone	Not applicable	Healthy animals	Improved drug delivery to bone-marrow space	[102]
